# Direct oral anticoagulants versus vitamin K antagonists for the treatment of venous thromboembolism in adults with sickle cell disease: a Bayesian meta-analysis

**DOI:** 10.1016/j.rpth.2026.107015

**Published:** 2026-10-14

**Authors:** Sylvain Le Jeune, Isabelle Mahé, Antonio Morabito, Jérémy Datchanamourty, Florent Happe, Edouard Versini, Louis Affo, Corinne Frere

**Affiliations:** 1 Department of Internal Medicine, Avicenne Hospital, Assistance Publique-Hôpitaux de Paris, Bobigny, France; 2 Université Sorbonne Paris Nord, Créteil, France; 3 INSERM U955, Institut Mondor de Recherche Biomédicale, Créteil, France; 4 F-CRIN INNOVTE network, Saint-Etienne, France; 5 Department of Internal Medicine, Louis Mourrier Hospital, Assistance Publique-Hôpitaux de Paris, Colombes, France; 6 Université Paris Cité, INSERM UMR_S970, Cardiovascular Research Center, Paris, France; 7 Department of Hematology, Pitié-Salpêtrière Hospital, Assistance Publique-Hôpitaux de Paris, Paris, France; 8 Sorbonne Université, INSERM UMR-S-1166, Institut Hospitalo-Universitaire ICAN, Paris, France

**Keywords:** bleeding, direct oral anticoagulants, sickle cell disease, venous thromboembolism, vitamin K antagonists

## Abstract

**Background:**

Direct oral anticoagulants (DOACs) are increasingly used as alternatives to vitamin K antagonists (VKAs) for the treatment of venous thromboembolism (VTE) in patients with sickle cell disease (SCD). However, comparative evidence is limited to small observational studies, and prior frequentist meta-analyses may be unreliable due to sparse data and zero-event outcomes.

**Objectives:**

This study aimed to compare the efficacy and safety of DOACs versus VKAs for VTE treatment in adults with SCD.

**Methods:**

We conducted a systematic review and Bayesian meta-analysis of studies comparing DOACs with VKAs in patients with SCD and VTE. PubMed, Embase, and CENTRAL were searched from inception to March 2026. Primary outcomes were recurrent VTE and major bleeding (MB). Bayesian random-effects models with weakly informative priors were used to estimate pooled odds ratios (ORs) with 95% credible intervals (CrIs). Posterior probabilities were derived using Hamiltonian Monte Carlo sampling.

**Results:**

Four retrospective studies including 271 patients (155 DOACs and 116 VKAs) were included. Recurrent VTE did not differ between groups (OR, 0.82; 95% CrI, 0.38-1.75), with a 67.1% posterior probability favoring DOACs, indicating equipoise. DOACs were associated with a significantly lower risk of MB (OR, 0.25; 95% CrI, 0.07-0.80), with a 99.2% probability of superiority. Between-study heterogeneity was low (τ = 0.15 for recurrent VTE; τ = 0.30 for MB).

**Conclusion:**

DOACs demonstrate comparable efficacy to VKAs for preventing recurrent VTE in SCD, with a clinically meaningful reduction in MB. Given their safety profile and convenience, DOACs may be preferred; randomized trials are needed to confirm these findings.

## Essentials

• Direct oral anticoagulants (DOACs) are increasingly used for thrombosis in sickle cell disease (SCD).
• Four studies compared DOACs with vitamin K antagonists (VKAs) in 271 adults with SCD.
• DOACs showed similar efficacy to VKAs in preventing recurrent venous thrombosis.
• DOACs reduced major bleeding, supporting a potential safety advantage over VKAs.

## Graphical abstract

**Figure:**
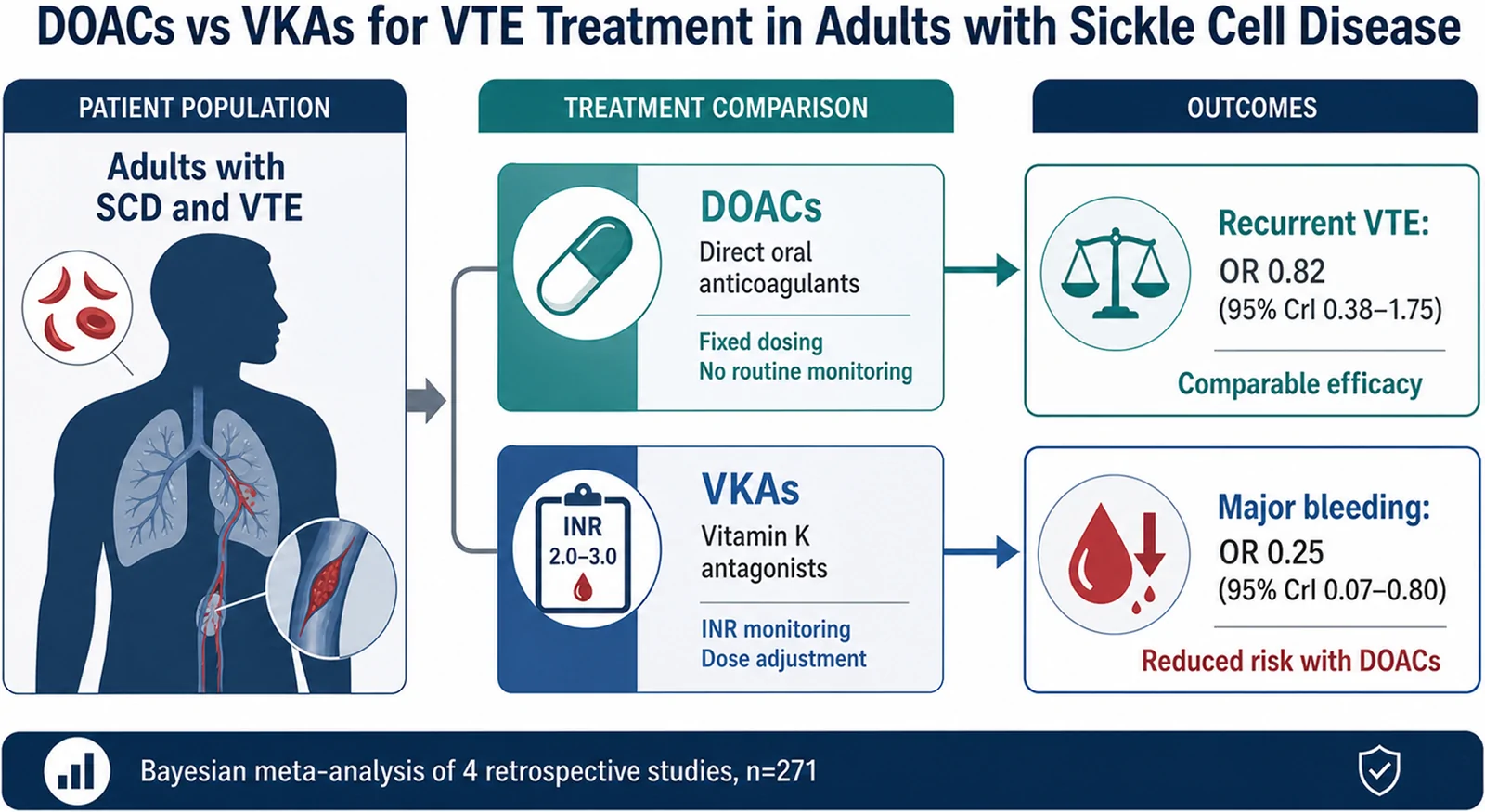

## 1 Introduction

Sickle cell disease (SCD) is an inherited hemoglobinopathy affecting millions worldwide, characterized by chronic hemolytic anemia, recurrent vaso-occlusive crises, and progressive vasculopathy resulting in end-organ damage [1]. Patients with SCD face a 3- to 10-fold higher risk of venous thromboembolism (VTE) than the general population, with a cumulative incidence of 11% to 12% by the age of 40 years [2], [3], [4], [5], [6]. The American Society of Hematology Clinical Guidelines for SCD suggest indefinite anticoagulation for patients with first unprovoked or recurrent provoked VTE, as SCD itself constitutes a chronic, persistent provoking factor for VTE [7]. Nevertheless, patients with SCD also face an increased risk of bleeding due to cumulative organ damage, notably gastrointestinal tract injury (use of nonsteroidal anti-inflammatory drugs, mucosal infarction from vascular occlusion, and stress ulceration from frequent hospitalization), cerebral vasculopathy, proliferative retinopathy, and chronic kidney disease, as well as frequent invasive procedures [8], which may influence anticoagulant choice and safety.

Although direct oral anticoagulants (DOACs) have been widely adopted for VTE treatment in SCD based on their favorable profile in the general population [9], [10], [11], [12], comparative evidence with vitamin K antagonists (VKAs) in this population remains scarce. A previous systematic review and meta-analysis identified limited, low-quality data comparing DOACs with VKAs in patients with SCD and acute VTE, with serious-to-critical risk of bias across included studies [13]. Traditional frequentist meta-analyses may lack robustness given small sample sizes and frequent zero-event data. Bayesian methods offer distinct advantages by providing probabilistic estimates of treatment effect, naturally accommodating sparse data, and explicitly quantifying uncertainty. We therefore conducted a Bayesian meta-analysis of observational studies comparing the efficacy and safety of DOACs versus VKAs for the treatment of VTE in adults with SCD.

## 2 Methods

This meta-analysis was prospectively registered with the International Prospective Register of Systematic Reviews (PROSPERO) (CRD42025177577) and conducted in accordance with the Preferred Reporting Items for Systematic Reviews and Meta-Analyses (PRISMA) guidelines [14]. We systematically searched PubMed, Embase, and the Cochrane Library (CENTRAL) from inception to March 19, 2026, for studies comparing DOACs with VKAs for VTE treatment in patients with SCD. The search strategy combined terms related to (1) SCD, (2) VTE, and (3) anticoagulants (see the Supplementary Table for the full search strategy). No language restrictions were applied. Reference lists of included studies were manually searched. Two authors (S.L.J. and C.F.) independently screened titles and abstracts, reviewed full texts, extracted data, and assessed methodological quality. Disagreements were resolved by consensus or adjudicated by a third reviewer (I.M.). Eligible studies were observational studies comparing DOACs with VKAs in adults (≥18 years) with SCD and acute VTE. Case series, cross-sectional studies, and studies not reporting outcomes of interest were excluded. The primary efficacy outcome was recurrent VTE, defined as a new objectively confirmed event (deep vein thrombosis [DVT] or pulmonary embolism [PE]) during follow-up. The primary safety outcome was major bleeding (MB), defined according to the International Society on Thrombosis and Haemostasis criteria [15] or study-specific definitions. The methodological quality of the studies was assessed using the Risk Of Bias In Non-randomized Studies of Interventions (ROBINS-I) tool [16]. No study reported sufficiently granular data to permit subgroup analyses, especially concerning patients with hepatic or renal impairment.

We performed Bayesian random-effects meta-analyses. Pooled odds ratios (ORs) with 95% credible intervals (CrIs) were estimated for each outcome. Weakly informative priors were specified: normal (0, 1.5) on the log-OR scale for treatment effects and exponential (1) for between-study heterogeneity (τ). These priors allow a wide range of plausible effect sizes while regularizing extreme estimates in small samples. Posterior distributions were obtained via Hamiltonian Monte Carlo sampling with 4 chains of 4000 iterations each (2000 warm-up), ensuring conv ergence (Rˆ < 1.01). Posterior probabilities were calculated to provide clinically interpretable estimates: *P*(OR < 1.0) assessed the probability of treatment benefit, and *P*(0.8 < OR < 1.25) evaluated equivalence within a clinically relevant margin. For MB, *P*(OR < 0.5) was additionally calculated to assess the probability of a clinically meaningful benefit (>50% risk reduction). Between-study heterogeneity was quantified using the posterior median τ with 95% CrIs. Sensitivity analyses used skeptical priors centered on the null (normal [0, 0.5] on the log-OR scale). We also examined robustness to alternative priors for heterogeneity. All analyses were performed in R (version 4.3.2, brms package, R Foundation for Statistical Computing).

## 3 Results

The literature search identified 1345 records, of which 4 met the predefined inclusion criteria (Figure 1) [17], [18], [19], [20]. All 4 studies were retrospective cohort studies published between 2018 and 2026. Together, these studies enrolled 271 adult patients with SCD and acute VTE: 155 received DOACs (apixaban, rivaroxaban, edoxaban, or dabigatran) and 116 received warfarin. The study population was predominantly women (60.9%), with a median age ranging from 28 to 33 years. Follow-up periods ranged from 6 to 44 months. Study characteristics and outcomes are summarized in Table 1. All 4 studies were judged to have a serious-to-critical risk of bias (Supplementary Figure). Baseline characteristics differed across studies and treatment arms, raising the possibility of confounding by indication; factors that may have influenced treatment choice were not reported.

**Figure 1:**
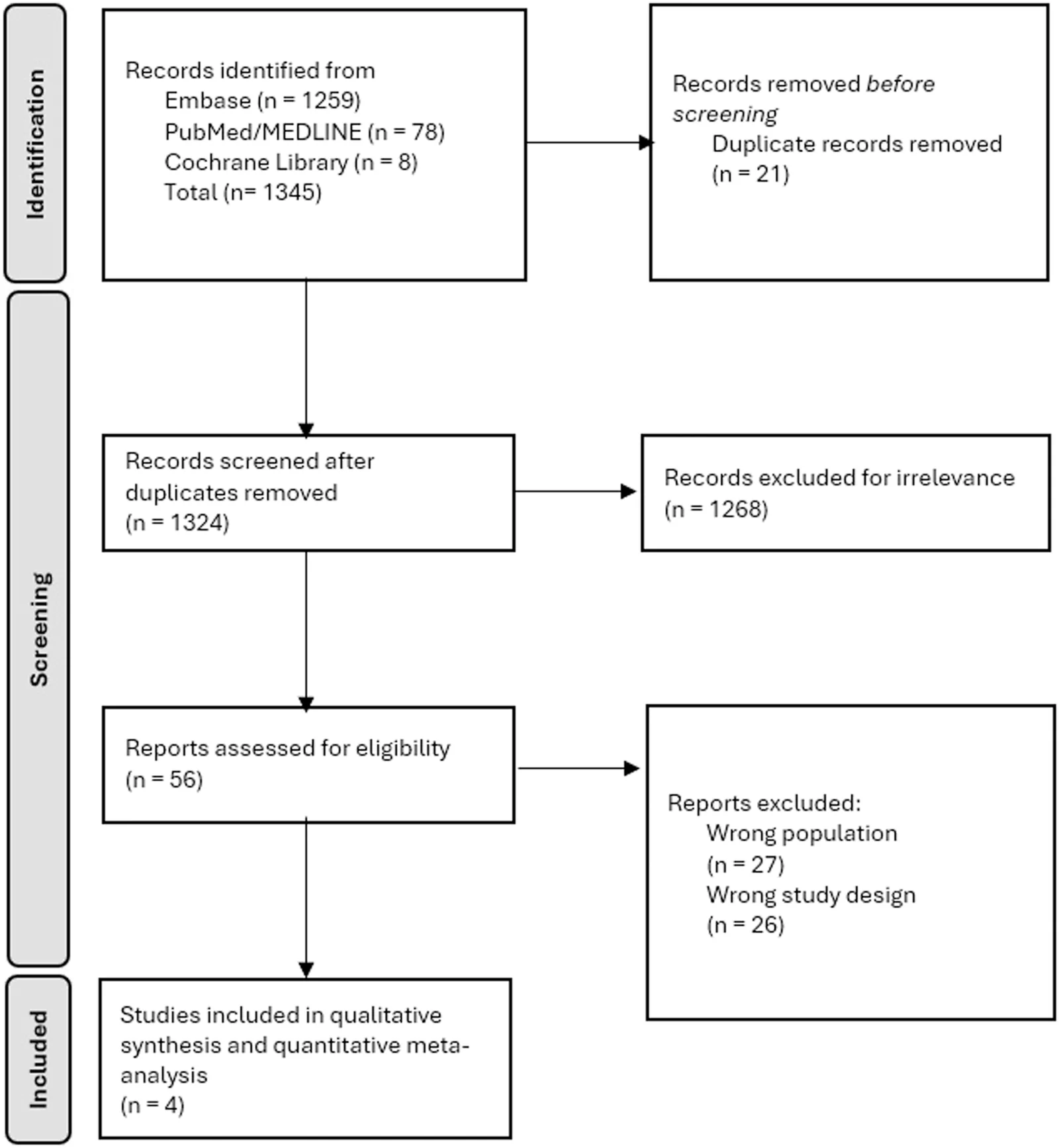
Preferred Reporting Items for Systematic Reviews and Meta-Analyses (PRISMA) flow chart of the selection process.

**Table 1:** Characteristics of included studies and participants.

Author, year	Study design	Location	Sample size, *n*	Index VTE type	Age (y)	Women, *n* (%)	Follow-up (mo)	Recurrent VTE, *n* (%)	Major bleeding, *n* (%)
Roberts et al., 2018 [17]	Retrospective cohort	United States	37	DVT: 22 PE: 15	Median: 28	20 (54)	6	DOACs: 6/22 (27.3) VKAs: 3/15 (20.0)	DOACs: 0/22 (0) VKAs: 1/15 (6.7)
Patel et al., 2018 [18]	Retrospective cohort	United States	75	DVT: 69 PE: 43^a^	Median: 33	49 (65.3)	11.8 (range: 3.4-60)	DOACs: 13/43 (30.2) VKAs: 10/32 (31.3)	DOACs: 2/43 (4.7) VKAs: 9/32 (28.1)
Gupta et al., 2018 [19]	Retrospective cohort	United States	60	DVT: 20 PE: 35^a^	NR	36 (65)	8.5 (IQR: 4.4-11.2)	DOACs: 4/23 (17.4) VKAs: 7/37 (18.9)	DOACs: 0/23 (0) VKAs: 2/37 (5.4)
Almegren et al., 2026 [20]	Retrospective cohort	Saudi Arabia, Oman, and Kuwait	99	CRT:5 CVT: 3 DVT: 26 PE: 64 Superior vena cava thrombosis: 2^a^ Portal vein thrombosis: 1 Budd-Chiari syndrome: 1	Mean: 32.8 ± 9.9	60 (60)	44 (range: 1-130)	DOACs: 4/67 (6.0) VKAs: 3/32 (9.4)	DOACs: 2/67 (3.0) VKAs: 0/32 (0)

CRT, catheter-related thrombosis; CVT, cerebral vein thrombosis; DOAC, direct oral anticoagulant; DVT, deep vein thrombosis; NR, not reported; PE, pulmonary embolism; SCD, sickle cell disease; VKA, vitamin K antagonist; VTE, venous thromboembolism.

a Numbers exceed total due to patients with combined events.

Recurrent VTE occurred in 27 of 155 patients (17.4%) receiving DOACs versus 23 of 116 patients (19.8%) receiving VKAs. The Bayesian pooled OR was 0.82 (95% CrI, 0.38-1.75) (Figure 2). The posterior probability that DOACs reduce recurrent VTE (*P*[OR < 1.0]) was 67.1%, indicating equipoise. The probability of equivalence (*P*[0.8 < OR < 1.25]) was 60.3%, supporting noninferiority. Between-study heterogeneity was minimal (τ = 0.15; 95% CrI, 0.00-0.52). Because the endpoint was analyzed as a binary patient-level outcome, these data do not address recurrent-event frequency or time-to-event differences.

**Figure 2:**
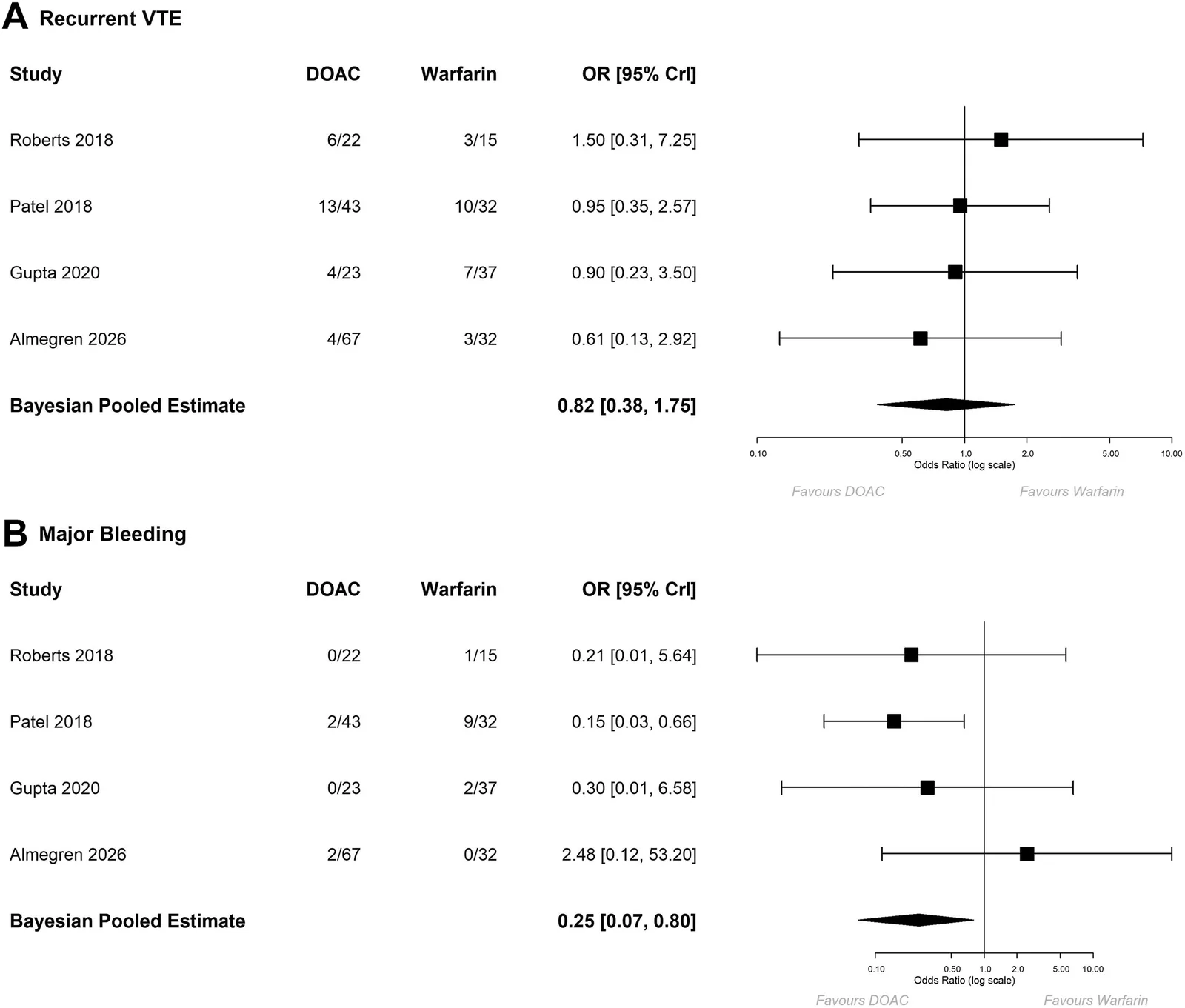
Bayesian hierarchical meta-analysis forest plots comparing direct oral anticoagulants to warfarin in sickle cell disease–associated venous thromboembolism (VTE). (A) Recurrent venous thromboembolism (VTE) and (B) major bleeding. The squares represent posterior median odds ratios (ORs) for individual studies, the horizontal lines represent 95% credible intervals (CrI), and the diamonds represent Bayesian hierarchical pooled posterior estimates. The vertical dashed line indicates no effect (OR = 1.0).DOAC, direct oral anticoagulant.

MB occurred in 4 of 155 patients (2.6%) receiving DOACs versus 12 of 116 patients (10.3%) receiving warfarin, corresponding to an absolute risk reduction of 7.7%. The Bayesian pooled OR was 0.25 (95% CrI, 0.07-0.80) (Figure 1). The posterior probability that DOACs reduce MB (*P*[OR < 1.0]) was 99.2%, providing strong evidence in favor of DOACs. The posterior probability of clinically meaningful benefit (*P*[OR < 0.5]) was 88.7%. Between-study heterogeneity was low (τ = 0.30; 95% CrI, 0.00-0.85). Three studies reported zero bleeding events in the DOAC or VKA groups [17], [19], [20], which Bayesian methods handle naturally without requiring *ad hoc* continuity corrections. However, this apparent safety advantage may still reflect residual confounding and treatment selection bias.

Sensitivity analyses using skeptical priors or evaluating the impact of heterogeneity priors (τ) confirmed the robustness of our findings (Table 2). Across all priors, the Bayesian pooled OR for recurrent VTE ranged from 0.80 to 0.86 (95% CrI, 0.37-1.82), with probabilities of equivalence (OR < 1) remaining stable between 65.3% and 68.5%. For MB, the OR remained consistently significant (0.22-0.30; 95% CrI, 0.06-0.90), with probabilities of benefit exceeding 98%.

**Table 2:** Sensitivity analyses evaluating the impact of priors for heterogeneity (τ) on model estimates.

Outcome	Prior (log OR)	Prior (τ)	OR (95% CrI)	P(OR < 1) (%)	τ (95% CrI)	WAIC
Recurrent VTE	Normal (0, 1.5)	Half-normal (0, 0.5)	0.82 (0.38-1.75)	67.1	0.15 (0.00-0.52)	120.5
	Normal (0, 0.5)	Half-normal (0, 0.5)	0.85 (0.40-1.80)	65.3	0.16 (0.00-0.55)	121.0
	Normal (0, 1.5)	Half-normal (0, 0.25)	0.80 (0.37-1.70)	68.5	0.12 (0.00-0.35)	122.1
	Normal (0, 1.5)	Half-Cauchy (0, 1)	0.86 (0.41-1.82)	66.2	0.28 (0.05-0.60)	121.3
Major bleeding	Normal (0, 1.5)	Half-normal (0, 0.5)	0.25 (0.07-0.80)	99.2	0.30 (0.00-0.85)	95.2
	Normal (0, 0.5)	Half-normal (0, 0.5)	0.28 (0.08-0.85)	98.5	0.28 (0.00-0.80)	96.0
	Normal (0, 1.5)	Half-normal (0, 0.25)	0.22 (0.06-0.75)	99.5	0.08 (0.00-0.25)	97.8
	Normal (0, 1.5)	Half-Cauchy (0, 1)	0.30 (0.09-0.90)	98.0	0.45 (0.10-1.00)	96.5

For recurrent venous thromboembolism (VTE), the pooled OR ranged from 0.80 (95% CrI, 0.37-1.70) under the skeptical prior for τ to 0.86 (95% CrI, 0.41-1.82) under the permissive prior, with probabilities of equivalence (OR < 1) remaining stable (65.3%-68.5%). For major bleeding, the OR remained consistently significant across priors (0.22-0.30; 95% CrI, 0.06-0.90), with probabilities of benefit exceeding 98%. Heterogeneity estimates (τ) varied modestly across priors (eg, 0.12-0.28 for VTE and 0.08-0.45 for bleeding) but did not alter the clinical interpretation of the results. The weakly informative prior (τ ∼ half-normal[0, 0.5]) provided the best model fit (lowest WAIC) for both outcomes.

CrI, credible interval; OR, odds ratio; WAIC, Watanabe-Akaike Information Criterion.

## 4 Discussion

This is the first study providing a probabilistic assessment of the efficacy and safety of DOACs versus VKAs for VTE treatment in adults with SCD. DOACs significantly reduced the risk of MB (OR, 0.25; 95% CrI, 0.07-0.80; posterior probability 99.2%) while maintaining comparable efficacy in preventing recurrent VTE (OR, 0.82; 95% CrI, 0.38-1.75; posterior probability 67.1%). Given the observational design, high risk of bias, and limited sample size, these data support a cautious preference for DOACs in eligible patients rather than definitive superiority.

The MB rate observed with VKAs should be interpreted cautiously; nonetheless, previous limited retrospective data reported a comparable rate of approximately 7% [7], and several disease-specific and treatment-related factors, including vaso-occlusive mucosal injury, hemostatic dysregulation, hepatic and renal dysfunction, and concomitant nonsteroidal anti-inflammatory drug use [8], may further amplify bleeding risk in this population.

The reduction in MB with DOACs is clinically relevant, particularly in patients with SCD who may have additional bleeding-risk modifiers. The 7.7% absolute risk reduction translates to one MB event prevented for every 13 patients treated with DOACs, achieved without compromising efficacy.

Beyond pharmacologic differences, VKA therapy poses practical challenges in SCD, including the need for frequent International Normalized Ratio monitoring, which may be difficult in patients with poor venous access and inconsistent access to regular outpatient follow-up. These real-world barriers may reduce the feasibility of VKA therapy and strengthen the practical appeal of DOACs in this population.

The Bayesian framework offers several advantages over frequentist meta-analyses for rare diseases with limited data. First, probabilistic statements provide direct answers to clinical questions rather than testing null hypotheses. Second, Bayesian methods naturally handle zero-event data through binomial likelihood, avoiding arbitrary continuity corrections that can introduce bias into estimates. Third, CrIs explicitly quantify posterior uncertainty, reflecting both sampling variability and between-study heterogeneity. The wide CrI for recurrent VTE (0.38-1.75) accurately reflects the limited precision resulting from data collected from only 271 patients. Importantly, Bayesian precision does not remove bias in the source studies; it only quantifies uncertainty around that bias.

Several limitations should be acknowledged. First, all included studies were observational, introducing the potential for confounding. The calendar periods during which DOAC- and VKA-treated patients were enrolled were not uniformly reported across studies. If VKA-treated patients were predominantly recruited during earlier periods than DOAC-treated patients, temporal changes in clinical practice could have introduced an additional source of bias. Selection bias may also have occurred if clinicians preferentially prescribed DOACs to patients perceived to be at lower bleeding risk. Monitoring bias is also plausible, given the more intensive International Normalized Ratio surveillance required for VKAs. Second, the small sample size limits precision, as reflected by wide CrIs. Third, follow-up durations were heterogeneous (6-44 months) and relatively short in most studies, potentially resulting in late events being missed. Fourth, no subgroup data were available to assess the impact of SCD genotype, renal function, concurrent hydroxyurea use, or specific DOAC agent. Fifth, DVT and PE were not consistently reported by anticoagulant group in the included studies, and recurrent events were rarely stratified by VTE subtype. As a result, separate effect-size estimates for DVT and PE could not be calculated. Finally, data sparsity precluded hierarchical Bayesian modeling to identify optimal candidates for DOAC therapy.

Prospective randomized controlled trials comparing DOACs with VKAs in adults with SCD and VTE are warranted. Such trials should incorporate a 12- to 24-month follow-up period and include SCD-specific endpoints, such as the frequency of vaso-occlusive crises and progression of organ dysfunction, and prespecify stratification for renal impairment, liver disease, and other factors likely to influence anticoagulant choice and bleeding risk.

Until such trials are conducted, this Bayesian meta-analysis provides the most rigorous evidence available to inform clinical decision-making and supports a cautious preference for DOACs in patients with SCD without contraindications.
