# Supplementary material for: Direct oral anticoagulants versus vitamin K antagonists for the treatment of venous thromboembolism in adults with sickle cell disease: a Bayesian meta-analysis
Source: Res Pract Thromb Haemost. 2026 Oct 14;10(7):107015. doi: 10.1016/j.rpth.2026.107015 (PMC13769079; doi:10.1016/j.rpth.2026.107015)
Supplement: Supplementary file 1 [file mmc1.docx]

**Supporting Information**

**Contents**

[**Supplementary Tables**	2](#_Toc229058064)

[**Table S1: Detailed search strings used for each database and number of records retrieved.**	2](#_Toc229058065)

[**Supplementary Figures**	4](#_Toc229058067)

[**Figure S1. Quality assessment.**	4](#_Toc229058068)

# **Supplementary Tables**

## T**able S1: Detailed search strings used for each database and number of records retrieved.**

| **Database** | **Search string** | **Records retrieved** |
| --- | --- | --- |
| **PubMed/MEDLINE** | ("sickle cell disease"[MeSH Terms] OR "sickle cell anemia"[MeSH Terms] OR "sickle cell disease"[Title/Abstract] OR "sickle cell anaemia"[Title/Abstract] OR "sickle cell anemia"[Title/Abstract] OR "hemoglobin SC disease"[Title/Abstract] OR "haemoglobin SC disease"[Title/Abstract] OR "SCD"[Title/Abstract] OR "SCA"[Title/Abstract]) AND ("venous thromboembolism"[MeSH Terms] OR "thrombosis"[MeSH Terms] OR "pulmonary embolism"[MeSH Terms] OR "venous thrombosis"[MeSH Terms] OR "thromboembolism"[Title/Abstract] OR "VTE"[Title/Abstract] OR "deep vein thrombosis"[Title/Abstract] OR "DVT"[Title/Abstract] OR "pulmonary embolism"[Title/Abstract] OR "PE"[Title/Abstract]) AND ("anticoagulants"[MeSH Terms] OR "direct oral anticoagulant*"[Title/Abstract] OR "DOAC*"[Title/Abstract] OR "NOAC*"[Title/Abstract] OR "novel oral anticoagulant*"[Title/Abstract] OR "rivaroxaban"[Title/Abstract] OR "apixaban"[Title/Abstract] OR "dabigatran"[Title/Abstract] OR "edoxaban"[Title/Abstract] OR "betrixaban"[Title/Abstract] OR "vitamin K antagonist*"[Title/Abstract] OR "VKA*"[Title/Abstract] OR "warfarin"[Title/Abstract] OR "acenocoumarol"[Title/Abstract] OR "fluindione"[Title/Abstract] OR "coumarin*"[Title/Abstract]) | 78 |
| **Embase** | ('sickle cell anemia'/exp OR 'sickle cell disease':ab,ti OR 'sickle cell anaemia':ab,ti OR 'sickle cell anemia':ab,ti OR 'hemoglobin SC disease':ab,ti OR 'haemoglobin SC disease':ab,ti OR 'SCD':ab,ti OR 'SCA':ab,ti) AND ('venous thromboembolism'/exp OR 'thrombosis'/exp OR 'pulmonary embolism'/exp OR 'deep vein thrombosis'/exp OR 'thromboembolism':ab,ti OR 'VTE':ab,ti OR 'deep vein thrombosis':ab,ti OR 'DVT':ab,ti OR 'pulmonary embolism':ab,ti OR 'PE':ab,ti) AND ('anticoagulant agent'/exp OR 'direct oral anticoagulant*':ab,ti OR 'DOAC*':ab,ti OR 'NOAC*':ab,ti OR 'novel oral anticoagulant*':ab,ti OR 'rivaroxaban'/exp OR 'apixaban'/exp OR 'dabigatran'/exp OR 'edoxaban'/exp OR 'betrixaban'/exp OR 'rivaroxaban':ab,ti OR 'apixaban':ab,ti OR 'dabigatran':ab,ti OR 'edoxaban':ab,ti OR 'vitamin K antagonist*':ab,ti OR 'VKA*':ab,ti OR 'warfarin'/exp OR 'acenocoumarol'/exp OR 'warfarin':ab,ti OR 'acenocoumarol':ab,ti OR 'fluindione':ab,ti OR 'coumarin*':ab,ti) | 1259 |
| **Cochrane Library** | (sickle cell disease OR sickle cell anaemia OR sickle cell anemia OR hemoglobin SC disease OR SCD OR SCA) AND (thromboembolism OR VTE OR deep vein thrombosis OR DVT OR pulmonary embolism OR PE) AND (direct oral anticoagulant* OR DOAC* OR NOAC* OR rivaroxaban OR apixaban OR dabigatran OR edoxaban OR vitamin K antagonist* OR VKA* OR warfarin OR acenocoumarol OR fluindione OR coumarin*) | 8 |

# **Supplementary Figures**

## **Figure S1. Quality assessment.**
